# Process Evaluation of Teaching Critical Thinking About Health Using the Informed Health Choices Intervention in Uganda: A Mixed Methods Study

**DOI:** 10.9745/GHSP-D-23-00484

**Published:** 2024-12-20

**Authors:** Ronald Ssenyonga, Simon Lewin, Esther Nakyejwe, Faith Chelagat, Michael Mugisha, Matt Oxman, Allen Nsangi, Daniel Semakula, Sarah E. Rosenbaum, Jenny Moberg, Andrew D. Oxman, Heather Munthe-Kaas, Christine Holst, Margaret Kaseje, Laetitia Nyirazinyoye, Nelson Sewankambo

**Affiliations:** a Department of Medicine, College of Health Sciences, Makerere University, Kampala, Uganda.; b Department of Epidemiology and Biostatistics, School of Public Health, Makerere University, Kampala, Uganda.; c Institute of Health and Society, Faculty of Medicine, University of Oslo, Oslo, Norway.; d Centre for Epidemic Interventions Research, Norwegian Institute of Public Health, Oslo, Norway.; e Department of Health Sciences, Norwegian University of Science and Technology, Ålesund, Norway.; f Health Systems Research Unit, South African Medical Research Council, Cape Town, South Africa.; g Tropical Institute of Community Health and Development in Africa, Kisumu, Kenya.; h School of Public Health, College of Medicine and Health Sciences, University of Rwanda, Kigali, Rwanda.; i Faculty of Health Sciences, Oslo Metropolitan University, Oslo, Norway.

## Abstract

**Introduction:**

We designed the Informed Health Choices (IHC) secondary school intervention and evaluated whether it improves students’ ability to assess the trustworthiness of claims about treatment effects in Uganda. We conducted a process evaluation alongside a randomized trial to identify factors that may affect the implementation, fidelity, and scaling up of the intervention in Uganda. We also explored the potential adverse and beneficial effects of the intervention.

**Methods:**

We used mixed methods to collect, triangulate, and report data from a variety of sources. We observed at least 1 lesson in all 40 intervention schools. One teacher from each of these schools completed a teacher training evaluation form and lesson evaluation questionnaires after each lesson. We purposively selected 10 schools where we conducted a total of 10 focus group discussions with students and 1 with parents. We also conducted key informant interviews with policymakers (N=9), teachers (N=10), head teachers (N=4), and parents (N=3). We used a framework analysis approach to analyze the data.

**Findings:**

All participants in the process evaluation felt that the IHC intervention was needed, important, and timely. Students were motivated to attend class and learn the content because it spoke to their daily life experiences and their own challenges to decide what to do or believe when faced with health claims. The training workshop gave teachers the confidence to teach the lessons. The participating students demonstrated a clear understanding of the content and use of what was learned. The content improved both students’ and teachers’ appreciation of the critical thinking, communication, and problem-solving competencies in the lower secondary school curriculum.

**Conclusion:**

The findings of this process evaluation are consistent with the findings of the trial, which showed that the intervention improved the students’ critical thinking skills. The IHC resources enabled teachers to teach this competency.

## Abstract (precis)

The Informed Health Choices educational resources improve students’ ability to critically appraise claims about the effects of health interventions. The resources also enable teachers to teach and assess critical thinking and problem-solving competencies using health as a topic.

## Teaser

**Key Findings:**

- Students, teachers, head teachers, and policymakers valued the lessons and recognized their importance in helping people cope with an overload of health information and in making informed health choices. Both students and teachers could relate to the health issues that the lessons addressed.
- Finding time to teach the lessons impeded their delivery. It may also affect scaling up the intervention. Factors, such as a busy school term that included sports, drama, and patriotic clubs, meant moving lessons around. This often led to teaching 2 lessons in 1 week rather than once a week as planned.
- The lessons were not in the curriculum and were not nationally examined. This may have affected teachers’ preparation for lessons, students’ attendance, and head teachers’ prioritization of the lessons.

**Key Implications:**

- All participating stakeholders recognized the need to be able to assess the reliability of health claims and make informed choices and appreciated the intervention. This may improve the chances of scaling up the intervention.
- However, scaling up the intervention may require incorporating the Information Health Choices lessons in the curriculum and national examinations.

See related articles by Mugisha et al. and Chesire et al.

## BACKGROUND

People need to deal with health information coming from multiple sources. This includes claims about the effects of health interventions made by health professionals, charlatans, politicians, journalists, advertisers, family members, and friends. Many health claims we are exposed to are misleading or unreliable, including claims found in traditional news media and on social media.^1^^,^^2^ When people believe unreliable claims, they may end up using ineffective or harmful interventions, which can result in unnecessary suffering and wasted resources.^3^^–^^7^ Conversely, when people fail to believe and act on reliable claims, this can also result in unnecessary suffering and inefficient use of resources.^8^^,^^9^

Critical thinking is key to making well-informed decisions and is seen as essential in a democracy.^10^ A good place to start building critical thinking about health (critical health literacy) is among young people, who, when in school, have time for learning and can bring knowledge back to their family members and community.^11^ There is a large population of young people in Uganda.^12^ We have an opportunity to equip that population with the skills they need to assess health claims and make informed personal choices and choices for others.

The Informed Health Choices (IHC) key concepts are principles that people need to understand and apply when deciding which health claims to believe and what to do. One example of a key concept is: “it should not be assumed that treatments are safe or effective or that they are not: treatments can cause harms as well as benefits.”^13^ We used an iterative process with key stakeholders to reach a consensus on selecting 9 (of 49) IHC key concepts to include in learning resources for secondary schools.^14^ We conducted an overview of systematic reviews of strategies for teaching critical thinking relevant to young people.^15^ We also conducted context analyses in Kenya, Rwanda, and Uganda to inform resource development and help ensure that they would be fit for the contexts in which they will be used.^16^^–^^18^ Using a human-centered design approach, we developed the IHC secondary school educational intervention, “Be smart about your health.”^19^^,^^20^ This was an iterative process with cycles of idea generation, prototyping solutions, piloting, user testing, and making improvements. Students, teachers, and curriculum developers provided feedback and input into the design of the resources.^21^

The IHC key concepts are principles that people need to understand and apply when deciding which health claims to believe and what to do.

The details of the intervention are described elsewhere^22^ and are summarized in the Box and in Supplement 1 using the Guideline for Reporting Evidence-based practice Educational interventions and Teaching checklist.

BOX Summary of the Informed Health Choices Secondary School Intervention

**Goal:** To give secondary school students a basic ability to think critically about health actions (things that people do to care for their health or the health of others); to understand why thinking critically is important; to be able to recognize claims about the effects of health actions; and to assess some of those claims.

**Underlying theory:** We used the Informed Health Choices (IHC) key concepts framework as a starting point for this educational intervention. These concepts or principles are intended to improve people’s ability to make better decisions on what to believe and do when faced with health claims and choices. The framework is based on evidence of the importance of the included concepts, logic, feedback, and other relevant framework.

**Content and planned delivery:** The intervention included:

- 2–3-day training workshop for secondary school teachers, facilitated by other teachers, to introduce them to the IHC learning resources and the learning content.
- 10 lessons for students in a single school term, with each lesson taught in 40 minutes.
- Overview and background for teachers for each of the 10 lessons, designed to be delivered using either a blackboard or a data projector. The 10 lessons were delivered by the teachers during regular classroom time or, if necessary, outside of regular classroom time. They could use a computer, smartphone, or printouts to support delivery of the lessons.

**Delivery:** Depending on what equipment was available to the teachers, teachers delivered the lessons to students using only a blackboard or a data projector and slide presentations that are included in the digital resources. The number of students in a class varied. Teachers used teaching strategies, such as guided note-taking, small group discussion, use of response cards, homework, use of a standard lesson structure, setting objectives, and providing feedback.

We conducted cluster randomized trials in East Africa (Uganda, Rwanda, and Kenya) to evaluate the effectiveness of the IHC secondary school educational intervention, comparing students in schools receiving the intervention to those in schools following the usual curriculum.^22^^–^^24^ The primary outcome was the proportion of students with a passing score on a test that measured their ability to think critically about health choices.^25^ In Uganda, 55.1% of students in schools that taught the IHC lessons had a passing score, compared to 24.7% in schools that did not.

We conducted this process evaluation alongside the randomized trial with 3 research objectives: to document the extent to which the intervention was delivered as intended, to identify factors that might affect the impact and scaling up the intervention in Uganda, and to explore potential adverse and beneficial effects of the intervention.

## METHODS

We carried out a mixed methods study using both qualitative and quantitative approaches. We collected qualitative data through classroom observations, key informant interviews (KIIs), and focus group discussions (FGDs) and quantitative data using training workshop evaluations and lesson evaluation forms. We used a logic model to link the process evaluation findings to the trial findings.^23^^,^^26^

### Qualitative Data Collection and Analysis

RS (principal investigator and PhD candidate with course credits in qualitative research methodology for doctoral students) and trained research assistants with a minimum of a bachelor’s degree collected data through KIIs, FGDs, and structured notes from the classroom lesson observations as well as the teachers’ evaluation forms (Supplement 2). Characteristics of the participants in the FGDs and KIIs are summarized in Table 1.

**TABLE 1.** Characteristics of Participants in Informed Health Choices Intervention Process Evaluation, Uganda

	**Data Collection Method (No.)**	**Total, No.**	**Sex,** **No. females**	**Age, Years, Median (IQR)**	**No. From Public Schools**
Students	FGD (10)	103	66	16 (15–17)	49
Teachers	KII (10)	10	6	32 (29–35)	5
Head teachers	KII (4)	4	2	49 (42–58)	2
Parent/guardian	FGD (1), KII (3)	11	7	43 (33–51)	9
Policymakers (curriculum developers, examinations director, national and district education officers)	KII (9)	9	1		N/A

Abbreviations: FGD, focus group discussion; KII, key informant interview; IQR, interquartile range; N/A, not applicable.

We used a framework thematic analysis approach to identify and compare themes across and within cases (Supplement 3).^27^ We have described the applied frameworks in detail in a separate protocol.^26^ We followed the stages of familiarization, coding with the identified thematic framework, charting, and interpretation of the data. Two of the researchers (RS and Joseph Balisanyuka (JB), a qualitative researcher with a master’s degree and qualitative research training) independently read and reread the transcripts, then coded the data using the factors included in the framework. Additionally, they searched for other factors. RS, JB, ADO, and SL then reviewed summaries of the coded data and considered additional factors suggested by the data. Disagreements during coding and charting were resolved by discussion. We charted the data by writing a summary of the findings for each framework factor. Then, we considered the extent to which the quantitative data supported those findings. Lastly, we explored possible explanations for the findings. We present the potential adverse outcomes in a separate article.^28^ All data were analyzed with the aid of Atlas.ti software version 9.

RS kept reflexive notes and participated in a team reflexivity process, where some of the co-authors wrote their reflections on expectations of the process evaluation findings and on how their background and experience might have shaped the work. Those notes informed 2 team reflexivity discussions. Key themes emerging from these reflections included issues related to the effects of the intervention, project sustainability and scaling up, the scope of the evaluation, the researchers’ relationship to the project and to the participants, and dynamics within the research team. These reflexivity discussions informed our analysis and write-up of the process evaluation. We describe the team reflexivity process as well as the key themes emerging from this in more detail in Supplement 4.

### Participant Recruitment

We purposively sampled 10 schools from each of these 4 strata: public school ownership versus government school ownership; schools that used a projector to teach the resources versus schools that did not use a projector. One head teacher from each of the 4 strata groups consented to school participation. Teachers at the selected schools helped us to identify parents who were willing to participate. All teachers who participated in teaching the intervention in the 10 selected schools were included in the research. From each of these 10 schools, we drew a random sample of 20 students from the attendance list of those who had taken the critical thinking about health test that was administered in the trial. From this sample, we selected consecutively the first 9–12 students to participant in FGDs. We also recruited the district and national officers in the secondary school education office from the districts where we implemented the trial, the examination board director, and 2 curriculum developers to participate in the KIIs. The process evaluation was conducted over a period of 15 weeks: 2 weeks before the school term that included teacher training, 10 weeks covering the teaching of the lessons, and 3 weeks of post-intervention implementation interviews.

### Quantitative Data Collection and Analysis

All teachers from the 40 schools in the intervention group of the trial completed training workshop evaluation forms. They also completed lesson evaluation forms after each delivered lesson (Supplement 2). In these forms, teachers reported students’ attendance, suitability of the resources for teaching, time it took to prepare and deliver each lesson, and what teaching strategies were used.^15^^,^^26^ Teachers used a Likert scale to rate their level of confidence to teach the lesson (1-not confident, 5-very confident), their preparedness (1- very unprepared, 5- very prepared), and ease of lesson delivery (1- very difficult, 4-very easy). We used electronic forms designed with SurveyCTO for the teacher training and lesson evaluations and exported data to STATA version 16 for generating descriptive statistics.

### Assessment of Confidence and Certainty in the Process Evaluation Findings

Two researchers (RS and JB) assessed the confidence in the findings using a modified version of the Grading of Recommendations Assessment, Development, and Evaluation Confidence in the Evidence from Reviews of Qualitative research (GRADE-CERQual) approach.^29^ For each finding, they assessed methodological limitations, coherence, adequacy, and relevance; then, they made an overall assessment of confidence in that finding (high, moderate, low, or very low). This approach is similar to that used in a process evaluation linked to the IHC primary school trial^30^ and in the Ugandan context analysis.^16^

### Link Between the Process Evaluation and Trial Findings

We summarized findings for each process evaluation objective and described how each finding may have influenced the outcomes of the trial. We also explored additional beneficial effects. We integrated the key findings into a logic model that includes intervention characteristics, effect modifiers, and short- and medium-term impacts.

### Participant Involvement Statement

We collected structured feedback from a wide range of stakeholders: students, parents, teachers, headteachers, and policymakers (curriculum developers, an examination official, and Ministry of Health and district educational officers). The processes to prioritize key concepts^14^ and develop the resources involved key stakeholders (teachers, children, and policymakers). They contributed ideas for the resources through consultative workshops and individual feedback through user testing and piloting of earlier versions of the resources.^20^^,^^21^

### Ethical Approval

We received ethical approval from the School of Medicine Research Ethics Committee at the Makerere University College of Health Sciences and from the Uganda National Council for Science and Technology. We obtained informed consent from head teachers on behalf of the school and students and from teachers for participation in the trial. We obtained informed consent from adult participants in FGDs and KIIs and informed assent from students who participated in FGDs. We also published a protocol before implementation.^26^

## RESULTS

We have integrated the main findings into a logic model in Table 2. We have summarized our assessments of confidence in those findings using the GRADE-CERQual approach in Supplement 5. We assessed that we had high confidence in all but 2 findings, which were rated as moderate confidence, which are indicated in the text. We report the findings for each of the study objectives.

**TABLE 2.** Logic Model Linking Information Health Choices Intervention Process Evaluation Findings to Randomized Control Trial Findings, Uganda

**Intervention Characteristics**	**Effect Modifiers**	**Short-Term Impacts**	**Medium-Term Impacts**
**Accessibility and adaptability of lessons:** Teachers found the IHC resources easy to access and adaptable (similar teaching strategies to those in the new curriculum, lesson structure easy to follow). (high confidence)	**Teacher training:** Teacher training improved understanding, motivation, and confidence among teachers to deliver the IHC lessons. (high confidence)	**Understanding of key concepts:** Most students were able to understand and apply the key concepts. Several students gave illustrations of how they applied them to think critically about health. This was mainly for concepts about claims and less for concepts about research. (high confidence)	This will be explored in the 1-year follow-up study.
**Value of the lessons:** All the students, teachers, head teachers, and policy makers interviewed valued the lessons and recognized their importance. (high confidence)	**Student motivation:** Most students found the lessons enjoyable, understandable (simple English, familiar examples) and related to the health issues that the lessons addressed. (high confidence)	**Interest in STEM subjects and health:** Several students expressed increased interest in STEM subjects and the health profession. Teachers also noticed this interest among some of their students. (moderate confidence)	
**Lesson delivery:** Nearly all planned IHC lessons were taught. Recommended teaching strategies and anticipated time for preparation (30 minutes) were used. However, few lessons were delivered as scheduled (once a week, during normal class time and within 40 minutes). Most lessons were taught in students’ private reading time, and all took longer than 40 minutes to deliver. (high confidence)	**National curriculum and examinations:** Because the IHC lessons were not in the curriculum and not nationally examined, teachers’ preparation for lessons, students’ attendance, and head teachers’ prioritization of the lessons were limited. (high confidence)	**Perceived benefit to students:** Students felt they were getting skills they would use now and even after school. (high confidence)	
**Resource credibility:** Credibility of the institution that developed the resources may have impacted effective delivery. Some student and teachers mentioned that they viewed material from Makerere University as important to learn. Other teachers mentioned the presence of curriculum developers at the teacher training workshop improved their trust of the resources. (high confidence)	The content was not assessed in the national examinations, yet the examinations remain a key motivator for use of additional learning resources in schools. Head teachers must decide what can be taught that contributes to the students’ scores. Teachers are often acknowledged based on their students’ performance on national examinations. Students take additional learning materials that are not examinable less seriously. (high confidence)	**Perceived benefit to teachers:** Teachers reported to have used the taught IHC concepts to access health information and make choices in their own lives. (high confidence)	
**Lack of time:** Finding time to teach IHC lessons impeded delivery as intended and may also affect scale-up of the intervention. (high confidence)			
**Need for printed materials:** Some policymakers and teachers expressed the need to have printed materials alongside the digital materials, particularly if the intervention was to be scaled up. For some, this was due to ICT challenges and how printed materials are used in the new curriculum. For others, this was to give students easy access to the material. (moderate confidence)			

Abbreviations: ICT, information communication technology; IHC, Informed Health Choices; STEM, science, technology, engineering, and math.

### Extent to Which the Intervention Was Delivered as Intended

Almost all participants (students, teachers, parents, and policymakers) who provided the data we present in this section did not have English as their first language.

All teachers used the teaching strategies we suggested. These were derived from an overview of systematic reviews of strategies for teaching critical thinking.^15^ They included class discussions, small group work, buzz groups, use of response cards, and guided note-taking across lessons. They also included concept-mapping, concept cartoons, inquiry-based instruction, quizzes, and role play for some lessons. We did not recommend debates but observed occurrences. Some teachers reported using debates in some lessons, especially in Lessons 8 (Personal choices) and 9 (Community choices).

#### Time Used to Teach the Lessons

We planned for the intervention to include 10 40-minute lessons that were to be taught once a week in a single school term. We anticipated that teachers would take 30 minutes to prepare for each lesson and that students’ attendance rates for the IHC lessons would be similar to other subjects like biology, English, and mathematics.

As anticipated, teachers spent an average of 32 minutes (standard deviation [SD] 12 minutes) preparing for the IHC lessons (Table 3). One teacher reported that he prepared for one of the lessons during the taxi ride to school. Teachers who felt that preparation took too long (over 40 minutes) highlighted that some of the resources were lengthy and needed to be read several times. In addition, there were other school engagements that interfered with the preparation time. Most of those who felt it took too long to prepare thought that preparation time would be shorter if they were teaching these lessons for a second or third time. Most teachers (93.5%) reported that they were prepared to teach the lessons, and 80% found they felt confident to teach the IHC lessons (Figure).

**TABLE 3.** Informed Health Choices Lessons Taught, Time Spent, and Student’s Attendance, Uganda

**Lesson**	**Schools, No.**	**Time Spent, Minutes, Mean (SD)**		**Students Attended IHC Lessons, Mean (SD)**
		**Preparation**	**Teaching**	
1: Health actions	40	31 (12)	67 (22)	71 (21)
2: Health claims	40	32 (8)	69 (14)	73 (23)
3: Unreliable claims	40	34 (11)	74 (12)	78 (20)
4: Reliable claims	40	34 (16)	71 (14)	74 (25)
5: Using what we learned	40	29 (11)	66 (16)	74 (26)
6: Randomly created groups	39	36 (11)	72 (17)	68 (23)
7: Large enough groups	39	32 (14)	69 (17)	68 (26)
8: Personal choices	39	30 (15)	67 (13)	73 (23)
9: Community choices	39	32 (12)	70 (14)	76 (35)
10: Using what we learned (2)	39	30 (14)	69 (18)	73 (27)
Overall		32 (12)	69 (16)	73 (25)

Abbreviation: SD, standard deviation.

Source of data: Teachers lesson evaluation forms.

**FIGURE:**
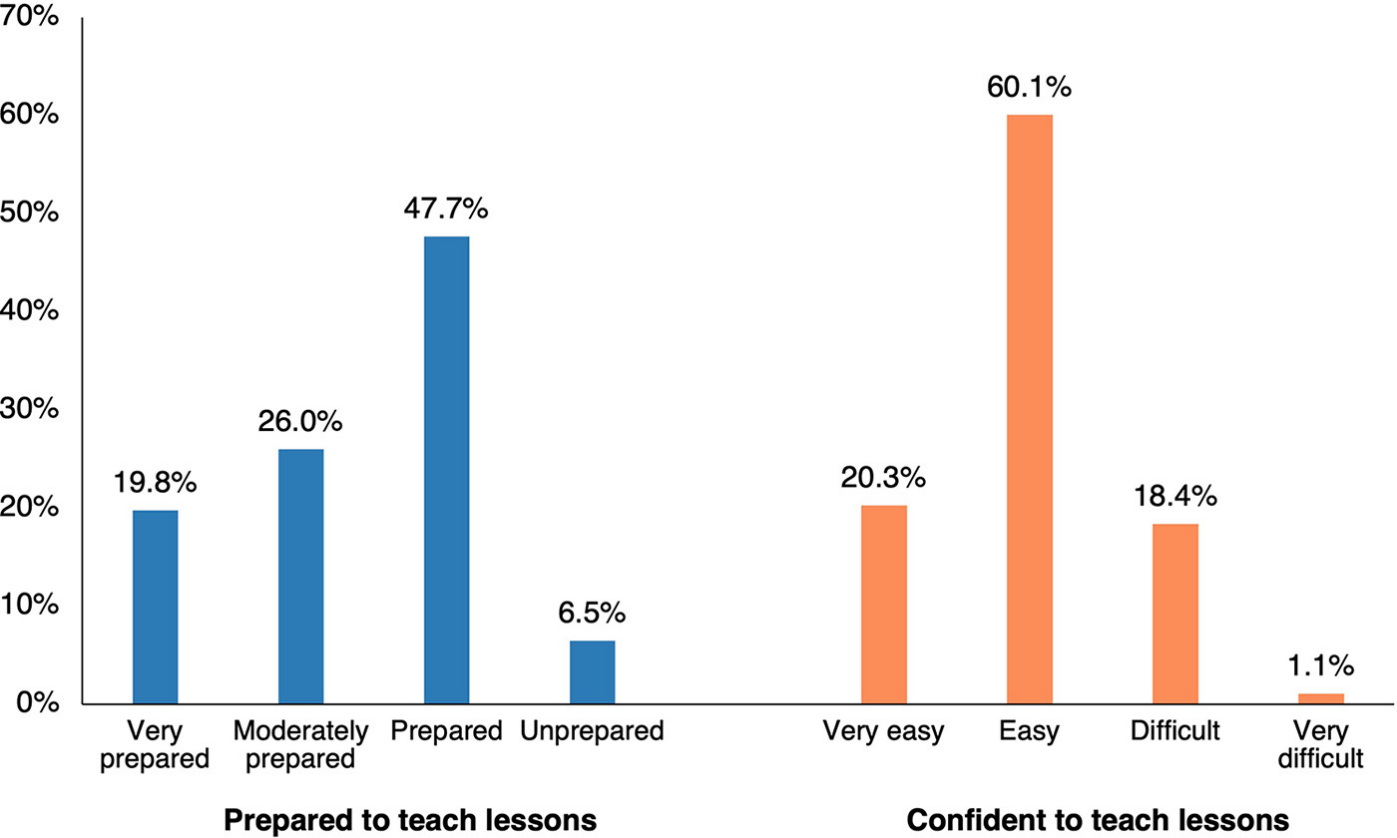
Teacher’s Feelings of Being Prepared and Confident to Teach the Informed Health Choices Lessons, Uganda

However, the delivery of IHC lessons took longer than we anticipated (Table 3). Despite repeated efforts during intervention development to simplify and shorten the lessons so they would fit into a 40-minute slot, lessons took, on average, 69 minutes to complete, and some took up to 80 minutes). Lessons 1 (Health actions) and 8 (Personal choices) took the least time, an average of 67 (SD 13) minutes. Lesson 3 (Unreliable claims) took the most time, an average of 74 (SD 12) minutes. The time used to teach these lessons was similar to what teachers initially recommended in the context analysis^16^ and what we found in a pilot study.^20^

Teachers carried out nearly all the planned lessons (395/400 [98.8%]). One teacher taught only 5 of the 10 lessons, citing a change in the school program that did not allow her to complete the lessons. Twenty-two of the 40 intervention schools were able to teach 1 lesson per week as planned. The remaining schools needed to teach more than 1 lesson a week due to missed lessons. Fourteen (90%) of the 16 schools with a projector either used it for all or most of the lessons. Class attendance among students fluctuated from lesson to lesson (Table 3).

Teachers carried out nearly all the planned lessons.

Despite an overly full school timetable after the long school closure due to the COVID-19 pandemic, all but 1 of the teachers were able to squeeze in the lessons. Nonetheless, this and several other factors may have adversely affected teachers’ and students’ motivation, the effort they put into these lessons, and what they got out of them. There also was a teachers’ strike that lasted 3 weeks at the beginning of the school term in which the intervention was delivered. In addition, this term included several extracurricular school activities, such as sports, music, and drama, as well as religious festivities, patriotic club trainings, and others.

*Students came back more mature [after a 2-year school closure due to COVID-19], less interested in academics, and also they faced many interruptions during the lessons, for example, some had to go for sports, drama, and academic tours.* —Teacher, public school, KII

#### Achievement of Lesson Objectives

Thirty-six of the 40 teachers reported having achieved the lesson objectives and mentioned that they used the number of correct responses from students to the wrap-up questions in the IHC lessons as a measure. This corresponded with our lesson observations. In FGDs, students correctly defined key terms and provided correct summaries of what they had learned. Teachers also reported this.

*Learners were able to define the terms like health actions, health claims and also provide a correct summary of what they had learnt from class, for example, identify helpful and harmful health actions, and also appreciate the use of critical thinking in their daily lives.* —Teacher, public school, teachers’ lesson evaluation forms

#### Accessing Resources

Only 1 of the 40 teachers did not use a smartphone to access the educational resources. This teacher’s phone was stolen after the teacher training workshop, so we provided him with a print-out of all 10 teacher summaries for the lessons. These 4–5-page summaries were part of the extra resources on the website and included the lesson goals, key terms, and the blackboard version of the lesson plan.

#### Delivery Description

We observed 67 lessons, with at least 1 lesson observed in each of the 40 schools that implemented the intervention (Table 4). In 97% of the observed lessons, teachers reviewed key messages from the previous lesson. They often asked students to say what they remembered from the previous lesson before the review. Our observations regarding students’ attendance, time spent delivering the IHC lessons, and teaching strategies did not differ from what teachers reported in the lesson evaluation forms. We observed that teachers tended to use class discussions rather than small group discussions or buzz groups when there was limited time to complete a lesson. This was also confirmed by teachers in interviews.

**TABLE 4.** Observations of Teachers’ Delivery of Informed Health Choices Lessons, Uganda

**Did teachers facilitate lesson delivery as intended?**	**Lesson Section and Corresponding Desired Delivery, No. (%) (N=67)**			
	**Introduction**		**Activity**	**Wrap-up**
	Teacher reviewed key messages from previous lesson	Teacher explained answers from review questions	Most students had opportunity to participate in activities	Teacher repeated key messages and asked students to take notes
Yes	48 (71.6)	66 (98.5)	0 (0.0)	62 (92.5)
Yes, for the most part	17 (26.4)	0 (0.0)	58 (86.6)	0 (0.0)
Yes, to some degree	2 (3.0)	0 (0.0)	0 (0.0)	0 (0.0)
No	0 (0.0)	1 (1.5)	9 (13.4)	5 (7.5)

Source of data: Lesson observation forms.

### Potential Desirable and Undesirable Effects of the Intervention

#### Perceived Overall Value

The intervention was aimed at equipping young people with skills and knowledge that could help them cope with the large amount of information available about the effects of health interventions. Several students mentioned that the IHC lessons improved their confidence to pause and reflect before deciding what to believe. A student reported that she used what she learned to help her cope with conflicting advice about health actions.

Several students mentioned that the IHC lessons improved their confidence to pause and reflect before deciding what to believe.

*You find people telling you different things, for example, someone tells you use lemon and another person tells you why don’t you use Colgate [toothpaste], another person tells me use avocado because my sister uses avocado, so you find yourself getting stressed but after going through these be smart [IHC] lessons, I realized that I am not supposed to get so much stressed on what people tell you to use. I will find something that works for me by using the things [principles] we have learnt.* —Student, FGD

It was evident from the lesson observation notes, the teachers’ lesson evaluation forms, interviews, and FGDs that this content is not taught in the curriculum, a finding that is consistent with results from our context analysis.^16^ Both the students and teachers appeared to appreciate the value of this additional educational content, expressing their inquisitiveness and a need to know how to teach and learn critical thinking skills.

*When it was just introduced, I thought that I knew everything because we youth always pretend to know more. Teacher had just introduced health. I was like I know my health; I will just attend but without writing. Then [we] reached a moment when he introduced claims, and they were divided into 2: reliable and unreliable. I realized I know nothing.* —Student, FGD

#### Learning Experiences Related to Individual Principles

As part of the intervention (Supplement 1 and Supplement 6), we introduced health actions (things that people do to care for their health or the health of others) and health claims (something that someone says as if it is true, but it may be wrong, about what will happen if you take a certain health action). Students listed many claims they had heard on the television, radio, online, on social media, or from friends or family. They also expressed that, before the IHC lessons, they had difficulties in deciding what to believe or do and that they were stressed by the overabundance of health information.

The 2 principles mentioned most often by students and teachers were: (1) the importance of weighing the advantages and disadvantages of health actions when deciding what to do, and (2) that personal experiences alone are a weak basis for claims about the effects of health actions. Teachers observed that students would highlight these 2 principles in other classes, and students reported these concepts in FGDs.

*I learnt [from the IHC lessons] not to do things just because others are doing them. For example, if [student003] tells me to use something because it worked for him, I would remember that just because it worked for him does not mean it will work the same for me. I will also think, what if it harms me instead of helping me?* —Student, FGD

The IHC lessons included 3 principles that intended to help people identify weak bases for health claims. These included claims based on personal experiences alone, on something being new or expensive, or on something being widely used or used for a long time. In both lesson observations and FGDs with students, several students explained how they applied these principles to claims that they had heard.

*For example, a health claim that taking lemon juice cures flu – is it reliable or non-reliable? Then you find out it’s non-reliable because it has a missing basis. Yet, I thought it was reliable [for others too] because I have tried it out several times and it worked for me. Later, I found out that because it worked for me, it might not work for my friend, which is a weak basis. [For] a claim to be reliable, [it] must have a strong basis like be approved by health practitioners and with research by scientists through making randomized trials.* —Student, FGD

*At home, our paternal family, most of them are doctors, so whenever there was a strong disease and then they call the doctor, they used to tell us to buy only expensive drugs, that expensive drugs are better. Through these [IHC] lessons, I realized that even cheap drugs can work.* —Student, FGD

The lessons were intended to help people question the basis of claims, recognize untrustworthy claims, and decide which treatment claims to believe and what to do. Several students applied the learned concepts to COVID-19 health interventions.

*My parents support the opposition [political party] so they said that when you get that [COVID-19] vaccination, you won’t be able to elect in the year 2026 [national presidential elections] because you will be dead. And I replied that, you mean the president does not want his supporters [to be alive]. I also asked, have you seen anyone dying because of this vaccine? Then they answered yes, and I asked again how sure are you it was the vaccine and are they more than those that vaccine has saved [protected]? They said they didn’t know, and I told them that I checked and the number of those cured [protected] is many. Then they said we are going but are afraid …They went and came back. A month passed, and they were fine.* —Student, FGD

The resources also taught people to be cautious of small studies, that the people being compared should be similar, and that random allocation was the best way to ensure this. Some students demonstrated they were able to recognize fair and unfair treatment comparisons when evidence was used to support a treatment claim.

*For unreliable claims, just because something is new, costly/expensive or is being used by many people doesn’t mean that it is reliable. In order for one to know that it is a reliable claim, health researchers have to first carry out research on 2 groups that are large enough and compare them… researchers should take part in creating these groups so they should be created randomly.* —Student, FGD

*We have to think about the claims we make or hear. Like in holidays, I met a man who was selling herbal medicine like emumba [herbs mixed with clay] saying they prevent cancer. Now, if I was not studying “Be smart” [IHC] lessons, because I learnt to look at the basis of the claim, I would have bought them. I had to deny them because he could not explain very well how those herbs could cure cancer and how he knew without comparing to other medicines.* —Student, FGD

Another principle that was taught in the intervention was for people not to assume that treatments were safe. A student reported how this helped decide about a treatment for pimples.

*My friends told me that, there was a new tube which cures pimples, and I remembered what the teacher told us while he taught critical thinking, that even though a new or expensive medicine/drug comes on the market you don’t have to just buy it, you have to first get some information that shows [it] is better and also know whether this thing can cure or cause you more harm on your face, and I decided to leave that medicine.* —Student, FGD

#### Other Perceived Benefits of the Intervention

Students, teachers, and parents reported several benefits from the IHC intervention. Students reported increased interest in learning, especially in relation to science, technology, engineering, and math subjects (moderate confidence). In addition, several students mentioned that they now aspire to become doctors or health researchers. Some teachers noticed a difference in their students’ desire to learn even in other subjects. Some teachers mentioned that the teaching strategies used in the resources improved their appreciation of the proposed strategies in the new curriculum.

*They [fellow teachers] said that these learners nowadays have more interest towards sciences and have developed a positive attitude towards learning. We have been looking for ways to encourage these learners … so these [IHC] lessons can motivate these learners.* —Teacher, public school, KII

*Ever since I started teaching these lessons [to these learners], their interest in biology increased. Some of them started saying that they wanted to be doctors, their attitude improved.* —Teacher, private school, KII

*This term our children have changed, especially mine. When I look at her … you can see she is very interested in studying. She said that this term they have exposed them to many things which she may not have seen.* —Parent, FGD

*You ask yourself when am I becoming a doctor because if you know biology or chemistry it helps you to be a doctor and I got it [interest] from these [IHC] lessons. After learning this [IHC concepts], I am feeling compelled to become one because a number of people come to our communities deceiving/defrauding people with the wrong medicine.* —Student, FGD

Students showed improved confidence in expressing their ideas to their peers, teachers, and parents. Teachers and parents observed some changes in student behavior that they attributed to the lessons. For example, teachers reported seeing increased engagement and reasoning in a group of students who were usually quiet before the IHC lessons. One of the parents mentioned that she saw the student in a drama skit leading scene, which she had never seen the child do before, and attributed this confidence to the IHC lessons.

Teachers and parents observed some changes in student behavior that they attributed to the lessons.

The principles included in the lessons are applicable in other fields besides health, such as education, agriculture, and people’s social lives. Students were also able to apply what they had learned in different contexts, for example, balancing advantages and disadvantages.

*There is a time we come to do health practices. For instance, I engage in sweeping this room. Yes, I am cleaning the room, but you have to look into the importance [advantages] and disadvantages because after cleaning, yes, the room will be clean, but I might end up with [a stuffy nose] from the dust as I sweep. So, you may find out that the disadvantages are more than the advantages. At times, because it is compulsory, we just take a risk and say let me just sweep the [room].* —Student, FGD

#### Potential Undesirable Effects of the Intervention

We reported the details of potential adverse outcomes associated with this intervention based on participants’ own perceptions and our observations in a separate qualitative evidence synthesis of the 3 trials.^28^ The most common potential undesirable effects we found were partial understanding and misunderstanding of the concepts.

### Factors That Might Have Affected the Impact of the Intervention

#### Perceived Value

Nearly all the students, teachers, and parents in FGDs and interviews could relate to the problem of overabundance of information about the effects of health interventions. They found it difficult to decide what to believe and do. Consequently, students, teachers, and policymakers recognized the importance of the lessons and valued them. The examples, content, and objectives of the lessons motivated the students to attend and actively participate in the lessons.

*Since it [IHC lessons] had common examples, like the things they have heard of or things they have seen in [their] community but don’t know what is behind them like the adverts on TV, someone coming up and telling you my product is the best for you. Even the examples [in the IHC lessons] were day-to-day things that we use, and sometime we believed them.* —Teacher, KII

*In other lessons you may find someone dozing, but in those [IHC] lessons, nobody was dozing. Everybody was active and even members from other streams were coming to attend with us … We are in a new curriculum that involves critical thinking, yet even us, we wanted to know that critical thinking in the new curriculum … when we heard that there was critical thinking in these [IHC] lessons, I was so motivated to go for these lessons.* —Student, FGD

*My daughter told me about a new topic, where they were teaching them how to think … this excited her because they were given a chance to speak their mind … for example, she would tell us what she learnt like deciding whether to believe that new medicine is better than the old medicine.* —Parent, FGD

#### Teacher Training

Another factor that might have positively influenced the intervention implementation was the teacher training workshop. All teachers reported in the workshop evaluation forms that they felt either confident or very confident teaching the IHC lessons after the 2-day teacher training workshop. However, in the interviews conducted after teaching the IHC lessons, some teachers reported feeling more confident teaching lessons about claims than those about research-related principles. Nonetheless, some teachers noted that the flow of the lessons made it easy for them and their students to understand the 2 lessons about assessing the trustworthiness of research evaluating the effects of health interventions.

*I felt confident to teach lessons on personal choices, community choices and even health actions and claims. Mainly those first lessons, and last. Also, the content was just logically sequenced, so that once a student understands from the [beginning] of the discussion, the other information is easy to follow.* —Teacher, KII

#### Flow of Lessons

Each lesson included a review of the previous lesson. Although several teachers found the review section good for the continuity in learning, a few teachers mentioned that if a student missed more than 1 lesson, they might not easily follow and comprehend the content of the lessons they missed.

*There is that logical flow of the [IHC] lessons. For example, I review the previous lesson content in every new class. Although, I see that if a student has missed more than 1 lesson in between, they find it difficult to follow.* —Teacher, KII

#### Absent From School Schedules, Curriculum, and National Exams

Several factors that may have hindered the impact and may also impede scaling up the intervention include the lack of allocated time to deliver the intervention and the intervention not being in the curriculum nor in the exams. The lessons were generally taught in place of students’ independent study time. Students found this inconvenient, especially when they learned that students in other streams and schools did not have these lessons.

Some students did not attend class when they learned that the IHC lessons were not taught in other streams and were not covered in the national exams. Even with the introduction of a competency-based curriculum in which a focus on examinations was discouraged, we found in the lesson observations, lesson evaluation forms, and interviews with teachers and students that teaching and learning still focused on exams. Teachers and students were less likely to spend time on lessons that were not included in the curriculum and exams, especially if these lessons took time away from subjects that were. We also observed that a few teachers did not seem to be well prepared when delivering the lessons, which may have affected the impact of the intervention. National examinations continue to be a key determinant of what students choose to study, what teachers choose to teach, and what school administrators choose to prioritize.

*My group members never liked the [first 2–3] lessons. At first, I was also among those who never liked the lessons, but the teacher kept going on [teaching]. I started liking the lesson but for them they never wanted it, they used to say, you are showing off, the things you are doing are not going to be set in UNEB [national exams] and I thought about leaving [the class], but I had to be bold and stay.* —Student, FGD

*I think again right now the exams are still important because they [student] use them to change schools but it is very clear in the new curriculum that we are emphasizing acquisition of generic skills.* —Policymaker, KII

#### Explanations in Local Languages

Students with low English proficiency reported that teachers and fellow students sometimes explained lesson content (e.g., definitions and instructions) in the local languages to improve understanding. This may have facilitated the impact of the lessons. Many students with low English proficiency attend under-resourced schools in rural areas. Thus, this strategy could potentially help to reduce inequities both within and between schools if the intervention is scaled up.

*The teacher came and told us about health claims. These are claims about health actions. It was new to me health claims. When my friend explained in Luganda [the local language], I realized I have been coming across those things but when I don’t know what health claims are. I got to know that health claims can be reliable and unreliable. That the reliable ones are those ones that can be trusted, and the non-reliable claims are those ones which you can’t rely on. I got to know that the claim has 3 parts, the basis, and claim and health action.* —Student, FGD

#### Easy to Access in Familiar Format

Teachers found the IHC digital lessons easy to access and presented in a familiar format to that in the new curriculum. This familiarity may have facilitated delivery of the lessons and their impact.

Teachers found the IHC digital lessons easy to access and presented in a familiar format to that in the new curriculum.

#### Potential Impact of Printable Materials on Effective Delivery

The IHC material included printable lesson summaries and posters, which may have increased the impact of the intervention. Most teachers that we interviewed preferred using their smartphones to access the lessons. However, we found that teachers still demonstrated a need for paper-based materials. For example, as mentioned earlier, 1 teacher requested printed lesson summaries upon losing his phone. We also observed 3 teachers using paper-based lesson summaries (printed at their respective schools) during the lessons, and 6 teachers made personal notes in a paper notebook to use as a back-up to their smartphones. A few teachers reported having to use personal notes because their phone’s screen froze during the lesson or they experienced a power outage during lessons taught using a projector. Furthermore, 3 teachers reported in interviews that they had printed lesson posters. Teachers and students reported finding the posters to be helpful when making personal notes and useful for students who had missed lessons.

Despite some teachers mentioning power outages during the school term, only 1 of the teachers reported that they had experienced challenges delivering lessons due to a power outage. Most mentioned the importance of adequate preparations, including ensuring fully charged phones before delivering the lessons and back-up hard copies of lesson summaries or personal notes.

Some teachers and policymakers expressed a desire for the entire set of printed resources to be provided (moderate confidence). They felt this would make it easier and quicker for both students and teachers to access and use the material. A few students also noted that like other subjects on the curriculum, the access to IHC printed resources in the school library would ease and improve their engagement with the resources.

### Factors That May Affect Scaling Up of the Intervention

#### Choice of School Term

The intervention may be better placed in a different school term. Some teachers suggested the first term of the year instead of the second or third term.

*First term would be the best, because, in second term, we are so much engaged with the candidates as we are preparing for mocks [exams] and there is a fight to cover the whole syllabus by second term. So, it becomes a bit of a challenge to have enough spare time [for] this entire program. But, first term the pressure is not much, the teaching load is also not much. So, we recommend first term.* —Teacher, KII

#### Relevance to Daily Life

Several students and teachers found the IHC lessons to be timely and beneficial, noting that the content related to their daily life experiences. This may motivate the students and teachers to continue engaging with this intervention and thus facilitate scaling it up.

*The [IHC] project is good, it is hands on. It is highly informative, participatory in a way that it arouses students’ capacity to think, relate with health information and practices, connect with everyday life. And most important is if you went through this quality of teaching, if [teachers] really had this package [the intervention] very well, it would help both themselves and the student make informed decisions about the way they live. And that is what counts in their life … In a typical rural school, you could see some life in the students at that school as a result of this project. … I think it can touch many lives of people because we are looking at students getting out of school with something beyond passing exams. We want to produce a holistic child.* —Policymaker, KII

#### Teaching Strategies That Facilitate Active Participation

During the activities section of the lessons, we observed that students were excited to participate in small groups and role-play. These teaching strategies and other elements of the intervention fit well with the new curriculum.

*Clearly, there were a number of students who were able to think critically. That one could be seen. There was communication taking place, which is among our [new curriculum] competencies. There was cooperation among the small group discussions, some form of teamwork. Then, like I said, there is also what we call self-directed learning – that individuals are using their own knowledge. They are not just receiving information from the teacher. …This is the kind of teaching approach that we want, where the learners construct their learning, the teacher plays the facilitating role and, yes, it really reflects on what NCDC [National Curriculum Development Committee] is advocating for and the [IHC] lessons fit well with what we are hoping to see as lessons in lower secondary schools.* —Curriculum specialist, KII

#### Perceived Credibility of Program Developers

The credibility of the institution associated with the intervention and how schools were approached may also have contributed to a positive reception and could potentially facilitate scaling up the intervention. Teachers at the training workshop reported that the presence of a government official from the curriculum office made it feel like a typical teacher training workshop that the government offers to introduce resources in the new curriculum. The credibility of Makerere University, letters of approval from the education ministry and district offices, and school consent by head teachers also contributed to teachers trusting the project and its objectives. Most students and teachers reported that they became interested when they heard that the educational resources were designed by researchers from Makerere University.

The factors influencing delivery and scale-up of the intervention are summarized in a logic model in Table 2. We highlight the intervention, immediate effects during implementation, and short- and medium-term impacts.

## DISCUSSION

### Facilitating Factors

Our randomized trial showed that the IHC secondary school intervention improved students’ ability to think critically and make informed choices.^22^ Consistent with those results, in this process evaluation, we found that students were able to understand and apply at least some of the key concepts that were taught. Students were able to apply the concepts in their daily lives. Teachers found the digital resources easy to access and adapt to their classrooms. Teachers also found the teaching strategies and lesson structure in the IHC lessons similar to those in the new lower secondary school curriculum. All the students, teachers, head teachers, and policymakers interviewed valued the lessons and recognized their importance. The teacher training improved understanding, motivation, and confidence among teachers to deliver the lessons. Students found the lessons enjoyable, understandable, and relevant. This motivated them and further motivated the teachers.

All the students, teachers, head teachers, and policymakers interviewed valued the lessons and recognized their importance.

Several factors related to our research strategies and choice of methods likely contributed to the positive reception of the resources and effectiveness of the intervention. First, together with teachers and curriculum specialists, we prioritized the concepts that were taught.^14^ This helped to ensure that the content of the lessons was appropriate and the learning goals were achievable. Second, our contextual analysis provided key insights into the conditions in the Uganda secondary schools.^16^ This helped to ensure that the resources were well suited for the context in which they were used and well aligned with the curriculum.^31^ Third, we used an iterative, human-centered design approach^32^^,^^33^ to develop the resources. This helped to ensure that they were fit for purpose, enjoyable, and understandable.^20^ We engaged teachers, curriculum developers, and students in developing the resources to ensure that they were easy to use, well suited for teaching in classrooms with limited ICT infrastructure, understandable, and relatable.

Students, teachers, head teachers, and policymakers all acknowledged that the IHC educational resources addressed important issues and imparted essential skills and knowledge for the 21^st^ century. These findings are similar to what was found in the process evaluation conducted alongside the primary school trial in Uganda.^34^ In addition, teachers reported that students’ interest in science, technology, engineering, and math subjects and their aspirations to become health professionals increased after the intervention. Teachers also became more confident in using the teaching strategies in the IHC resources to teach critical thinking,^15^ which were similar to those recommended in the new curriculum.^35^ To help facilitate sustainability and scalability, we developed the intervention together with the stakeholders in the education (commissioners of public and private schools, Lead of information communication and technology (ICT) in secondary schools), and health sectors (commissioner of health promotion, lead school health desk), including teachers, students, curriculum developers, and education officers. The intervention was designed to be adaptable to varied classroom settings so that in addition to the intervention being sustained among the schools that participated in the trial, it could also be extended to other schools in the country. To support this, at the end of the trial, we invited teachers in the control schools to a training workshop similar to that given to teachers in the intervention schools at the start of the trial. We also encouraged teachers from both intervention and control schools to continue teaching the lessons. For example, in Uganda, we also kept the teachers’ WhatsApp group active and continued to respond to questions and clarifications. This may, in the short term, support sustainability in the schools that participated in the trial. The project has attempted to address the scalability of the intervention in several ways. First, the intervention was designed to allow scale-up at relatively low cost by focusing on digital rather than paper-based training resources. Second, key country stakeholders, including the Ministry of Education curriculum department, were involved in the design of the intervention. Following the trial, we have engaged key stakeholders in the Ministry of Education in discussions on the future scale-up of the intervention, and these deliberations continue. In addition, we held a webinar including representatives from the curriculum and education offices of 3 East African countries to discuss scalability and opportunities to incorporate the intervention into the national curriculum, along with what remains to be done to gather more evidence around this intervention. We obtained commitments of support for the intervention from the 3 countries, and we continue engaging these officials.

### Impeding Factors

Because the IHC lessons were not in the curriculum, it was difficult for teachers to prioritize time for them. We also found that teaching the lessons took longer than we had planned, but largely, the implementation was delivered as intended. The challenge of finding time for material that is not in the curriculum is well documented.^16^^,^^36^ School administrators may lack motivation to allocate time for teaching, teachers may lack motivation to prepare adequately before delivering lessons, and students may lack motivation to attend classes. This is especially the case for material that is not included in national assessments. Schools in Uganda are still ranked from top to bottom based on performance on national exams, and the rankings are reported on television and in newspapers. Most schools admit students using prior academic scores and reward teachers based on student’s performance. Thus, incorporating the lessons in the curriculum and in national exams is likely to be important, if not essential, for scaling up and sustaining the intervention. Systematic reviews on the challenges of implementing competency-based curricula, as well as other topics, such as sexuality education, in secondary schools collectively highlight the multifaceted implementation issues that are encountered. This work emphasized the need for culturally sensitive approaches, comprehensive training for educators, supportive policies, and community involvement to successfully implement and sustain effective education programs.^37^

Lack of paper-based resources might be a barrier to wide-scale uptake. We designed these digital resources deliberately as non-paper based to avoid printing costs that can be prohibitive for schools in low-resource environments. We also took care to create resources that could be used offline in light of the limited ICT infrastructure in many schools.^16^ In Uganda, as in many parts of the world, the use of digital technology has increased dramatically over the last decade with an intentional drive to use ICT in learning and not only in administration and entertainment.^38^ The new lower secondary school curriculum encourages the use of ICT in learning.^37^ However, some students, teachers, and policymakers expressed a desire for hard copies and suggested that paper-based resources would be necessary if they were to be widely used, at least for some of the materials. Future iterations should explore in more depth the kind of paper-based resources that should be made available, for which user groups, and for use in what contexts.

### Strength and Limitations

We gathered data using a variety of methods, facilitating triangulation and strengthening our confidence in the findings. In addition, we used a modified CERQual approach to transparently assess confidence in our findings. Nine of the 11 results presented in the summary of findings (Supplement 5) were assessed as having high certainty, and the other 2 with moderate certainty.

A limitation of this study is that the team of investigators was responsible for both developing and evaluating the intervention. This may have led us to emphasize participants’ positive experiences. We emphasized to the participants that the IHC resources were being tested and not them. Nonetheless, there may have been some desirability bias. We used a team reflexivity process (Supplement 4) to mitigate these risks.

## CONCLUSION

The value and importance placed on the objectives and content of the IHC secondary school resources played a key role in delivering the intervention as intended in Uganda. Although there was limited time available to teach the lessons and these were not part of the curriculum or national exams, this did not appear to affect negatively the impact of the intervention. The new competency-based curriculum in Uganda aims to shift the focus away from lower-order thinking skills and “teaching to the test” to higher-order thinking skills. However, there is still a heavy focus on national exams that do not test higher-order thinking skills. Efforts to test higher-order thinking skills in national exams, as well as investment in developing and sustainably implementing effective and accessible educational interventions, like the Informed Health Choices secondary school intervention, are needed.

## Supplementary Material

GHSP-D-23-00484-Supplements.pdf

## Notes

**Peer Reviewed**
