# Supplementary material for: Process Evaluation of Teaching Critical Thinking About Health Using the Informed Health Choices Intervention in Uganda: A Mixed Methods Study
Source: Glob Health Sci Pract. 2024 Dec 20;12(6):e2300484. doi: 10.9745/GHSP-D-23-00484 (PMC11666090; doi:10.9745/GHSP-D-23-00484)
Supplement: GHSP-D-23-00484-Supplements.pdf [file GHSP-D-23-00484-Supplements.pdf]

Supplement to: Ssenyonga R, Lewin S, Nakyejwe E, et al. Process evaluation of teaching critical thinking about health using the Informed Health Choices Intervention in Uganda: a mixed methods study. *Glob Health Sci Pract*. 2024;12(6):e2300484. https://doi.org/10.9745/GHSP-D-23-00484

Supplement 1. Uganda IHC secondary school Intervention

GREET checklist

Guideline for reporting evidence-based practice educational interventions and teaching (GREET) checklist[1]

BRIEF NAME

1. **Intervention:** Informed Health Choices (IHC) secondary school intervention

The intervention was compared to routine practice (teaching according to the national lower-secondary school curriculum without intervening).

WHY this educational process

2. **Theory:** The IHC secondary school resources are based on the *IHC Key Concepts* framework. The framework includes concepts (principles) that people should understand and apply when deciding whether to believe a claim about the effects of health actions (things that people do to care for their health or the health of others) and what to do. [2,3] The framework is based on evidence of the importance of the included concepts, [4,5] logic, feedback, other relevant frameworks, [6] and adaptation of the IHC Key Concepts to other types of interventions such as educational, environmental, and policing interventions. [7]

The resources were developed by the investigators between 2020 and 2022 using human-centred design methods. [8] This included cycles of idea generation and prototyping, piloting with observation, user-testing with teachers and students, and feedback from teachers, students, and curriculum developers in Kenya, Rwanda, and Uganda, and an international advisory group. The aim of the design process was to ensure that teachers and students find the resources to be engaging, useful, and easy to use.

The teaching strategies used in the resources were based in part on an overview of systematic reviews of teaching strategies, [9] and draw on several educational theories. These include social constructivist theory (which postulates that learning can be maximized through well-designed, intentional social interaction with other learners), [10] the theory of active student response (which postulates that learning is enhanced by high levels of active student response), [11] and the elaborative retrieval hypothesis (which postulates that the search for correct answers on practice tests or quizzes results in multiple retrieval routes which aid later recall). [12]

3. **Learning objectives:** The primary learning goal is for students to have a basic ability to think critically about health actions and understand why this is important. They should be able to recognise claims about the effects of health actions and assess some of those claims. They should understand why it is important for them that researchers study the effects of health actions and recognise two key features of reliable comparisons of health actions. They should recognise that health actions can have both advantages and disadvantages and the importance of weighing the benefits and savings against the harms and costs when deciding what to do.

4. **Evidence-based practice content:** The resources focus on nine IHC Key Concepts that were prioritised by curriculum developers, teachers, and researchers in Kenya, Rwanda, and Uganda. [13]

WHAT

5. **Materials:** The IHC secondary school resources (*Be smart about your health*) are open access digital resources for lower-secondary school teachers. The 10 lessons are provided as lesson plans in two formats: for teachers who are using either a blackboard and or a projector in the classroom. The aim is for students to learn to think critically about health claims and choices. The resources were made available to schools in the intervention group. Teachers in those schools downloaded the resources to a computer or smartphone and delivered the lessons. Schools in both the control and intervention group continued teaching the national curriculum, which did not include teaching critical thinking about health. No additional materials were provided to the control schools.

Each Lesson includes an introduction, an activity, and a wrap-up. The introduction includes the key messages from the previous lesson, a question about the previous lesson, and what this lesson is about. The activity is designed to help students achieve the learning goals. The wrap-up includes a question about what was learned, the key messages for the lesson, a homework assignment, if there is one, and what the next lesson is about. Lessons 5 and 10 include quizzes and discussions of application of what students learned in their daily lives.

For each of the 10 lessons there is an overview and background for teachers. The overview includes learning goals, key terms introduced in the lesson, and the main teaching strategies used in the lesson. The background includes a description of what the lesson is about and if relevant, common misunderstandings and closely related content that is not covered in the lesson.

In addition, there is a teachers' guide, materials for teacher training workshops, information about how to use the resources (help), optional printouts (PDFs) for teachers and students, and a glossary. Teachers were provided with binders with printouts at the training workshops.

6. **Educational strategies:** Key strategies used across lessons included guided note taking, small group discussion, use of response cards, [11] homework, use of a standard lesson structure, setting objectives and providing feedback, and multimedia design. Other strategies used in some of the lessons include concept cartoons, inquiry-based instruction, and role play.

7. **Incentives:** The incentive for teachers and students was the value they perceived in learning to think critically about health actions. Teachers at schools without Internet access were reimbursed for the cost of downloading the resources and any other costs related to participation in the trial. They were not paid for participating in the trial and there were no other financial incentives for the schools, head teachers, teachers, or students. The evaluation administered at the end of the school term did not count towards the students' school marks or assessment of the teachers or schools.

WHO PROVIDED

8. **Instructors**: The head teacher at each participating school selected a teacher of a relevant subject (e.g., biology) for year-1 or year-2 of lower-secondary school. The teachers were invited to a 2-3-day workshop to introduce them to the resources and the learning content. The training was facilitated by other teachers who had participated in one of the teacher networks that helped to develop the resources or who piloted use of the resources. The facilitators were provided with presentations and other materials for the workshops, and they reviewed the material and plans for the workshops with the research teams prior to the workshops.

HOW

9. **Delivery:** The 10 lessons were delivered by the teachers during regular classroom time or, if necessary, outside of regular classroom time. They could use a computer, smartphone, or printouts

when delivering the lessons. Depending on what equipment was available to the teachers, they delivered the lessons to students using only a blackboard or using a projector and slide presentations that are included in the digital resources. The number of students in a class varied.

WHERE

10. **Environment:** Representative samples of schools were recruited, including rural and urban schools. The conditions in the schools varied. Details of the contexts in each of the three countries can be found in reports of the context analyses undertaken prior to developing the resources. [14-16]

WHEN and HOW MUCH

11. **Schedule:** The 10 lessons were taught in a single school term. Each school decided how to fit the lessons into the schedule for that term.

12. **Amount of time**: Each lesson is designed to be delivered in a single period (40 minutes). The students were encouraged to collect and assess claims about the effects of health actions outside of class and to discuss claims with their families and friends. The teachers needed up to 30 minutes to prepare for each lesson.

PLANNED CHANGES

13. **Adaptation**: No specific adaptation was required, but teachers were able to adapt the lessons, for example by using different or additional examples or editing the presentations.

UNPLANNED CHANGES

14. **Modifications**: As part of the process evaluations, teachers were asked to complete an evaluation form after each lesson, including information about changes they made to the lesson plan, and each teacher was observed for one lesson. No feedback was given to the teachers during the trial.

HOW WELL

15. **Attendance:** The teachers were asked to record attendance for each lesson. Students were encouraged to attend all lessons by telling them when the next lesson would be and its learning goals. The lessons were designed to appeal to students and to make clear the relevance and importance of the learning goals.

16. **Fidelity**: We will explore the extent to which the lessons were delivered as planned in the process evaluation, based on the evaluation forms completed by teachers after each lesson, observations of their teaching a lesson, and interviews with teachers and students.

17. **Delivery schedule**: The teachers were asked to record when each lesson was taught, the duration of each lesson, and whether all the lesson were completed as planned.

References

1. Phillips AC, Lewis LK, McEvoy MP, et al. Development and validation of the guideline for reporting evidence-based practice educational interventions and teaching (GREET). *BMC Med Educ* 2016; **16**: 237.

2. Oxman AD, Chalmers I, Austvoll-Dahlgren A, Informed Health Choices Group. Key Concepts for assessing claims about treatment effects and making well-informed treatment choices. *F1000Res* 2019; **7**: 1784.
3. Oxman AD, Chalmers I, Dahlgren A, Informed Health Choices Group. Key Concepts for assessing claims about treatment effects and making well-informed treatment choices. Version 2019. *IHC Working Paper* 2019.
4. Oxman AD, Chalmers I, Dahlgren A, Informed Health Choices Group. Key Concepts for Informed Health Choices: a framework for enabling people to think critically about health claims (Version 2022). *IHC Working Paper* 2022.
5. Oxman AD, Chalmers I, Dahlgren A. Key Concepts for Informed Health Choices: Where's the evidence? *F1000Res* 2022; **11**: 890.
6. Oxman AD, Martinez Garcia L. Comparison of the Informed Health Choices Key Concepts to other frameworks that are relevant to learning how to think critically about treatment claims, comparisons, and choices: protocol for a mapping review. *IHC Working Paper* 2018.
7. Aronson JK, Barends E, Boruch R, et al. Key concepts for making informed choices. *Nature* 2019; **572**(7769): 303-6.
8. Rosenbaum SE, Moberg J, Chesire F, et al. Teaching critical thinking about health information and choices in secondary schools: human-centred design of digital resources. *F1000Res* 2023.
9. Oxman AD, Nsangi A, Martinez Garcia L, et al. The effects of teaching strategies on learning to think critically in primary and secondary schools: an overview of systematic reviews. *Review of Education* Submitted 20 January 2023.
10. Igel C. Cooperative learning. Denver, CO: McREL International, 2010.
11. Randolph JJ. Meta-analysis of the research on response cards: effects on test achievement, quiz achievement, participation, and off-task behavior. *J Posit Behav Interv* 2007; **9**(2): 113-28.
12. Pan SC, Rickard TC. Transfer of test-enhanced learning: meta-analytic review and synthesis. *Psychol Bull* 2018; **144**(7): 710-56.
13. Agaba JJ, Chesire F, Mugisha M, et al. Prioritisation of Informed Health Choices (IHC) Key Concepts to be included in lower-secondary school resources: a consensus study. *PLoS One* 2023: in press.
14. Mugisha M, Uwitonze AM, Chesire F, et al. Teaching critical thinking about health using digital technology in lower secondary schools in Rwanda: A qualitative context analysis. *PLoS One* 2021; **16**(3): e0248773.
15. Ssenyonga R, Sewankambo NK, Mugagga SK, et al. Learning to think critically about health using digital technology in Ugandan lower secondary schools: a contextual analysis. *PLoS One* 2022; **17**(2): e0260367.
16. Chesire F, Ochieng M, Mugisha M, et al. Contextualizing critical thinking about health using digital technology in secondary schools in Kenya: a qualitative analysis. *Pilot and Feasibility Studies* 2022: Forthcoming.

Supplement to: Ssenyonga R, Lewin S, Nakyejwe E, et al. Process evaluation of teaching critical thinking about health using the Informed Health Choices Intervention in Uganda: a mixed methods study. *Glob Health Sci Pract*. 2024;12(6):e2300484. https://doi.org/10.9745/GHSP-D-23-00484

Supplement 2. Tools used for the data collection

Teachers' workshop evaluation forms 2
Web document 2: Lesson Evaluation Form 8
Web document 3: Lesson observation form........ 6
Web document 4: Interview guide 16

Supplement to: Ssenyonga R, Lewin S, Nakyejwe E, et al. Process evaluation of teaching critical thinking about health using the Informed Health Choices Intervention in Uganda: a mixed methods study. *Glob Health Sci Pract*. 2024;12(6):e2300484. https://doi.org/10.9745/GHSP-D-23-00484

Teachers' Workshop Evaluation Form

This form is to collect feedback on the workshop you have attended. Kindly give honest feedback to help us improve.	
School code:	
Teacher code	
Date:	

Please indicate your impressions of the items listed below.

**Training components**	Strongly Agree	Agree	Neutral	Disagree	Strongly Disagree
1. The training gave me general understanding of the critical thinking about health.	❍	❍	❍	❍	❍
2. The training gave me a clear overview and flow of all lessons.	❍	❍	❍	❍	❍
3. I can navigate through the resources, and I know where I can find all that I need on the website	❍	❍	❍	❍	❍
4. Now I understand all teaching strategies relevant for teaching critical thinking about health	❍	❍	❍	❍	❍
5. The training gave me teaching tips that I need to consider while teaching CHOICE lessons	❍	❍	❍	❍	❍
6. I am confident that I understand and can teach all 10 lessons	❍	❍	❍	❍	❍
**Competences**					
7. The training met my expectations.	❍	❍	❍	❍	❍
8. I will be able to apply the knowledge learned.	❍	❍	❍	❍	❍
9. The training objectives for each topic were identified and followed.	❍	❍	❍	❍	❍
**Training materials**					
10. The content was organized and easy to follow.	❍	❍	❍	❍	❍
11. The materials distributed were pertinent and useful.	❍	❍	❍	❍	❍

Supplement to: Ssenyonga R, Lewin S, Nakyejwe E, et al. Process evaluation of teaching critical thinking about health using the Informed Health Choices Intervention in Uganda: a mixed methods study. *Glob Health Sci Pract*. 2024;12(6):e2300484. https://doi.org/10.9745/GHSP-D-23-00484

Trainers

12. The trainer was knowledgeable.	❍	❍	❍	❍	❍
13. The quality of instruction was good.	❍	❍	❍	❍	❍
14. The trainer met the training objectives.	❍	❍	❍	❍	❍
15. Class participation and interaction were encouraged.	❍	❍	❍	❍	❍
16. Adequate time was provided for questions and discussion.	❍	❍	❍	❍	❍

17. How do you rate the training overall?

Excellent	Good	Average	Poor	Very poor	
❍	❍	❍	❍	❍	

18. What aspects of the training could be improved?

19. What was most useful?

20. What was least useful?

21. Other comments?

THANK YOU FOR YOUR PARTICIPATION!

Supplement to: Ssenyonga R, Lewin S, Nakyejwe E, et al. Process evaluation of teaching critical thinking about health using the Informed Health Choices Intervention in Uganda: a mixed methods study. *Glob Health Sci Pract*. 2024;12(6):e2300484. https://doi.org/10.9745/GHSP-D-23-00484

Web document 2: Lesson Evaluation Form

Instructions for teachers

This form is for you to:
1. Reflect on what you did well in preparing and teaching this lesson and how you could improve.
2. Provide feedback on the materials you used to teach today
3. Help us understand why learners did or did not achieve the lesson objectives

Please feel free to contact Ronald Ssenyonga on 0700733108 if you have any questions/challenges completing this form.

Section 1/3: Introduction

A1. School ID:	
A2. Teacher ID:	
A3. Lesson # Title:	
A4. Date:	
A4b. Roughly, how long did the lesson take (in minutes)? (*Please enter numbers only*)	

A5. Number of students in class today	
A6. How did you teach the lesson?	☐ Blackboard ☐ Projector
A6b. If you used a projector, did you use it for the entire lesson? (*If you used projector*)	Yes, for the whole lesson, Yes, for the most part in the lesson, Yes, but for the least part of the lesson
A7. Which teaching strategies & assessments methods did you use in this lesson?	☐ role play ☐ multimedia design ☐ small groups ☐ buzz groups<br>☐ class discussions ☐ response cards/ thumbs UP or Down ☐ debate ☐ other

Supplement to: Ssenyonga R, Lewin S, Nakyejwe E, et al. Process evaluation of teaching critical thinking about health using the Informed Health Choices Intervention in Uganda: a mixed methods study. *Glob Health Sci Pract*. 2024;12(6):e2300484. https://doi.org/10.9745/GHSP-D-23-00484

Section 2/3: Preparation

B1. How long did it take you to prepapre to for this lesson?	Very short (Less than 10 minutes), Short (20 minutes), Moderate (About 30 minutes), Long (1 hour), Too long (Over 1 hour)
B2. How would you rate your own preparation before the lesson?	Very unprepared, Unprepared, Moderately prepared, Prepared, Very prepared
B3. What made it easy or difficult for you to prepare for today's lesson? (*write your thoughts below*)	

Section 3/3: Teaching the lesson

C1. Did you manage to complete the lesson today?	Yes, No
C2. How easy or difficult overall was this [Lesson taught] for you to teach?	very difficult, difficult, easy, very easy
C2a. Give some reasons why you think it was easy or difficult overall to teach this lesson.	
C3. How would you rate the extent to which lesson objectives were achieved?	Didn't achieve, Not sure, Achieved
C4. Why do you think the lesson objectives were/were not achieved?	

Thank you for this feedback!

Supplement to: Ssenyonga R, Lewin S, Nakyejwe E, et al. Process evaluation of teaching critical thinking about health using the Informed Health Choices Intervention in Uganda: a mixed methods study. *Glob Health Sci Pract*. 2024;12(6):e2300484. https://doi.org/10.9745/GHSP-D-23-00484

Lesson Observation form

(5 page form, 1 page instructions)

Lesson # Title:	
Version:	☐ Blackboard ☐ Projector

Date:	
School ID:	
School type:	☐ Public ☐ Private ☐ Government-aided
Observer(s):	

Scheduled start/end time of lesson	
Number of students attending	
Number of teachers in class	
Type of technology teacher uses	☐ laptop ☐ smart phone ☐ computer lab ☐ projector ☐ other

See Instruction page: **‘Instructions before the observation starts’** and **‘Observation materials’**

Pre-lesson

B1. Record what the teacher has done before the lesson, including:<br><br>**For the blackboard version**:<br>Note if anything is written on the blackboard.<br><br>**For the projector version:**	

Supplement to: Ssenyonga R, Lewin S, Nakyejwe E, et al. Process evaluation of teaching critical thinking about health using the Informed Health Choices Intervention in Uganda: a mixed methods study. *Glob Health Sci Pract*. 2024;12(6):e2300484. https://doi.org/10.9745/GHSP-D-23-00484

Note whether the projector is set up and ready for use, has the lesson set up and is ready for use	

Equipment/technology used during lesson

Note the type of equipment/technology used.<br><br>**For the blackboard and projector version**:<br><br>Note if the students have response cards.<br><br>**For the projector version:**<br><br>Note whether the projector is set up and ready for use, has the lesson set up and is ready for use. Note the type of audio-visual equipment used: (smart phone/projector).<br><br>**For the projector and computer versions:**<br>Note if there are any power outages or loss of Internet connection during the lesson and how these are managed.	

Start of the lesson/timing

Planned start time of the lesson:		
Actual start time of lesson:<br>[Do not let teacher or students know you are timing lesson.]		
B2. If there is a substantial gap between the planned start time and when the lesson started, note what happened during that time.		
Keep track of whether more or less time is used for the quiz and review of the previous lesson (about 10 minutes), the activity (about 20 minutes), and the wrap-up (about 10 minutes).	B3a. Introduction	Enter actual time spent:
	B3b. Activity	Enter time spent:

Supplement to: Ssenyonga R, Lewin S, Nakyejwe E, et al. Process evaluation of teaching critical thinking about health using the Informed Health Choices Intervention in Uganda: a mixed methods study. *Glob Health Sci Pract*. 2024;12(6):e2300484. https://doi.org/10.9745/GHSP-D-23-00484

	B3c. Wrap up	Enter time spent:

See observer instructions: "During the lesson"

Quiz and review of previous lesson

Start time of the quiz:	
C1a. Did all the students respond?<br><br>C1b. Did the teacher explain the answers?	
C1c. Did the teacher review the key messages from the previous lesson?<br><br>C1d. Did the teacher check to see if there were any questions or misunderstandings?	

Lesson activity

Start time of the lesson activity:	
D1a. Did you observe that every students had an opportunity to participate in class? (Could be through buzz 0r small groups, class discussions etc. If yes, how or If no, how?)<br><br>D1b. Were the activities clear after teacher explanations?	
Lesson activity continued	

Wrap up & Observers impressions

Start time Wrap up	

Supplement to: Ssenyonga R, Lewin S, Nakyejwe E, et al. Process evaluation of teaching critical thinking about health using the Informed Health Choices Intervention in Uganda: a mixed methods study. *Glob Health Sci Pract*. 2024;12(6):e2300484. https://doi.org/10.9745/GHSP-D-23-00484

E1a. Did all the students respond to wrap up question(s)? Did the teacher explain the answers?<br><br>E1b. Did the teacher repeat the key messages and ask the students to make sure they have them in their notes?<br><br>E1c. Did the teacher give the assignment and information about the next lesson?<br><br>E1d. Did the teacher check whether the students had questions or misunderstandings?<br><br>F1a. Overall, teachers: how did the teacher appear to respond to the lesson?<br>*(Did they seem to enjoy it? Did the teacher get frustrated or bored? Did they say anything about the lesson?)*<br><br>F1b. Overall, students: how did the students appear to respond to the lesson? *(Did they seem to enjoy it? Did they seem engaged? Did they get frustrated or bored? Did you hear them saying anything about the lesson to eachother?)*<br><br>F1c. Overall, School environment: how did the school environment appear to facilitate the lesson delivery?	
Did she give the assignment and information about the next lesson?	
Did she check whether the students had questions or misunderstandings?	
G1c. Did you observe any adverse outcomes, or were there observations that might indicate an adverse outcome? (• *A student or teacher misunderstanding an explanation or example*<br>• *Conflict between students, students and teachers, or others*<br>• *Distraction due to irrelevant, excessive, or difficult questions from students*<br>• *Any other adverse outcome*)	

Supplement to: Ssenyonga R, Lewin S, Nakyejwe E, et al. Process evaluation of teaching critical thinking about health using the Informed Health Choices Intervention in Uganda: a mixed methods study. *Glob Health Sci Pract*. 2024;12(6):e2300484. https://doi.org/10.9745/GHSP-D-23-00484

G1d. Did you observe any transfer of learning, or were there observations that might indicate transfer of learning? (• *Transfer of learning to other fields, besides health*<br>• *Transfer of learning to practical choices about what to believe or do, in daily life*<br>• *Any other transfer of learning*)	
End time Wrap up	

Post lesson

Overall, teachers: how did the teacher appear to respond to the lesson?<br>(Did they seem to enjoy it? Did the teacher get frustrated or bored? Did they say anything about the lesson?)	
Overall, students: how did the students appear to respond to the lesson?<br>(Did they seem to enjoy it?<br><br>Did they seem engaged? Did they get frustrated or bored? Did you hear them saying anything about the lesson to eachother?)	

G. Please write down any other thoughts (*Include any other thoughts you have on the lesson observered today*)

Supplement to: Ssenyonga R, Lewin S, Nakyejwe E, et al. Process evaluation of teaching critical thinking about health using the Informed Health Choices Intervention in Uganda: a mixed methods study. *Glob Health Sci Pract*. 2024;12(6):e2300484. https://doi.org/10.9745/GHSP-D-23-00484

Instructions for observers

Instructions before the observation starts:

- Review the lesson plan before the lesson and bring a copy with you to follow along while observing the lesson.
- Share the study objectives. (Remind them that we are observing how the students and teachers interact with the materials)
- Explain the data collection methods we are using for the observation (non-participatory observation).
- Sit in the back of the class to ensure that there is no class distraction
- seek consent for their participation and to take pictures

Observation materials:

- Observation Guide (printed)
- Consent forms (printed copies to be signed by the teacher)
- Notebooks
- Pens/pencils
- Identification card (mandatory- if visiting study participant for the first time)
- Covid-19 PPE (masks, sanitizer etc)
- Voice recorder/camera

During the lesson

Follow along in the lesson plan, so you can note how the teacher uses and understands it, e.g., whether the teacher misunderstands or skips steps. Note things like:

- What seems to work well, or not well
- What the students and teacher seem to like or dislike
- What the students and teacher seem to misunderstand, e.g., an illustration, an example, or an activity
- Any words or concepts the teacher and students struggle with
- What examples the students and teacher use, other than those in the lesson
- If the teacher has problems using the resources, including any technical problems
- Note any deviations from the lesson plan or enhancements and whether they seemed to work well or were problematic
- Note whether the teacher told the students to take notes when indicated and whether the students took notes
- Anything else that you think is important for the effective use of the resources

Supplement to: Ssenyonga R, Lewin S, Nakyejwe E, et al. Process evaluation of teaching critical thinking about health using the Informed Health Choices Intervention in Uganda: a mixed methods study. *Glob Health Sci Pract*. 2024;12(6):e2300484. https://doi.org/10.9745/GHSP-D-23-00484

Interview guide

Process evaluation interviews	
Interview ID (type of school/respondent):	
Interview no:	
Date:	
Interviewer/ Observer:	
Audio recording (Yes/No)?	

BEFORE THE INTERVIEW: INTRODUCTION AND CONSENT

Introduce yourself and your role in the project, briefly

Refer to the information that the participant should have received via telephone and/or information meeting beforehand.

Inform the participant about their rights and our responsibilities.

- Data will be handled anonymously.
- Sensitive personal information will not be saved
- They are free to end the interview at any time, without giving a reason

Describe and explain the project, briefly, using plain language.

- There are many claims about what is good for our health.
- Many of those claims are unreliable (we cannot be sure that they are correct)
- Makerere University together with partners in other countries, are developing resources for secondary schools, to help students think critically (carefully) about health choices.

Explain the purpose of the interview, briefly:

- We want to learn from your experience, so the resources we develop are appropriate.
- You are not being tested, and there no wrong answers.

Request written consent to participation and to being recorded

Begin recording if given written consent to do so

Supplement to: Ssenyonga R, Lewin S, Nakyejwe E, et al. Process evaluation of teaching critical thinking about health using the Informed Health Choices Intervention in Uganda: a mixed methods study. *Glob Health Sci Pract*. 2024;12(6):e2300484. https://doi.org/10.9745/GHSP-D-23-00484

INTERVIEW

Ask the participant to introduce themselves (without revealing their identity)

Prompts for teachers/students

- Type of school ownership, class sizes
- Age of student, favorite subject

Objective 1: document the extent to which the be smart about your health intervention is delivered as intended.

T1a. To what extent did you deliver the "Be smart about your health" lessons as planned?

Prompts:

- In what time (40 or 80 mins)
- Teaching style
- Changes in the way you teach other subjects

T1b. What school activities did you have to fore-go/swap/replace in order to teach the "Be smart about your health" lessons?

Prompts:

- Do you think it was worthwhile for you and the students?

T1c. Did you feel that you managed to engage the students during the lessons and get them thinking and discussing, or was this difficult to do with these lessons?

Prompt:

- class size too large, children notused to discuss in class, children weretired/too early in the day, etc.
- **If yes:** Can you say something how orwhy you were able to get students engaged in these lessons? **If no:** Why was this difficult?

Objective 3: identify factors that affect effective delivery and scale up of the be smart about your health intervention in secondary schools.

T3a. What are your general thoughts about the "Be smart about your health" lessons?

T3b. What sort of skills do you think helped you teach the "Be smart about your health" lessons in an effective way?
Prompt: What skills or competencies did you feel you lacked?

T3c. What are your thoughts on the training you received in the delivery ofthe IHC lessons?

Prompts:

- Suggestions on what can be improved?
- Specific example(s) how you think the training helped you prepare to deliver the lessons.

Supplement to: Ssenyonga R, Lewin S, Nakyejwe E, et al. Process evaluation of teaching critical thinking about health using the Informed Health Choices Intervention in Uganda: a mixed methods study. *Glob Health Sci Pract*. 2024;12(6):e2300484. https://doi.org/10.9745/GHSP-D-23-00484

T3d. Tell me a bit about how you felt teaching this material to your class.

Prompts:

- Did you feel confident/certain?
- experience any conflicts (You & your beliefs - Can you give an example of akind of conflict you experienced with these resources)
- Did it change the way you approached lessons?

T3d. How motivated did you feel to teach these lessons to your class – very motivated or not so motivated?

Prompts:

- Why?
- Did you look forward to teaching these lessons? Or did you feel they were a burden somehow?

T3e. What needs to be done/improved in the current version of “Be smart about your health” lessons to ease use of these resources in all schools in Uganda.

Prompts:

- Do you think teachers can teach them with out any support?

T3d. What needs to be done by the policy makers at the ministry to incorporate the “Be smart about your health” lessons for use in all schools in Uganda.

T3e. How easy or difficult was the preparation to deliver the IHC lessons?

T3f. Can you tell us briefly how the students in your class responded to being taught these lessons?

Prompt:

- it can be positive or negativeresponses.

T3g. How easy or difficult was it for you to take on and to teach the “Be smart about your health secondary school resources” lessons in addition to all your other responsibilities at the school?

Prompt:

- Did you lack resources to carry outthe teaching effectively?
- Did you lack time in your schedule? Were theselessons competing for time that you feel might have been spent better doing other things?
- Was it a burden to prepare for lessons or tocorrect exercise books?

T3h. Besides time constraints, were there other factors that made it difficult to teachthese lessons in your school.

Prompt:

- lack of support/interest from your leaders
- lack of support/interest from your peers
- lack of support/interest from parents orcommunity
- School resources (human, equipment, etc)

Supplement to: Ssenyonga R, Lewin S, Nakyejwe E, et al. Process evaluation of teaching critical thinking about health using the Informed Health Choices Intervention in Uganda: a mixed methods study. *Glob Health Sci Pract*. 2024;12(6):e2300484. https://doi.org/10.9745/GHSP-D-23-00484

- Political environment
- Bureaucracy
- Incentives and disincentives

Objective 2: explore intended and unintended potential effects of the be smart about your health intervention among students, and teachers in lower secondary schools of Uganda.

T2a. Have you experienced or observed the lessons having any disadvantages? If so, please tell us about it.

Prompts:

- Misunderstanding
- Conflict
- Distraction
- Stress, or other uncomfortable thoughts or feeling
- Wasted time or resources
- Other

T2b. Do you have any suggestions of other possible good effects that these resources or learning these concepts might have on people?

Prompts:

- Examples from students or your self

T2c. Do you have any suggestions of other possible bad effects that these resources or learning these concepts might have on people?

Prompts:

- Examples from students or your self

Supplement to: Ssenyonga R, Lewin S, Nakyejwe E, et al. Process evaluation of teaching critical thinking about health using the Informed Health Choices Intervention in Uganda: a mixed methods study. *Glob Health Sci Pract*. 2024;12(6):e2300484. https://doi.org/10.9745/GHSP-D-23-00484

SUPPLEMENT 3. Framework for factors that could affect the implementation, impact, and scaling up of the school resources

Domain	Factors and sources	Explanation
Teachers	Skills and competencies	Teacher's education and experience in relation to the lessons being taught.
	Understanding of the content being taught	Teacher's understanding of the content.
	Sufficient training	The extent to which the teachers received sufficient training in teaching the lessons
	Self-efficacy	Teacher's confidence in teaching the lessons.
	Fit to the teacher's teaching style and context (eg, class size)	Teachers' comfort with the instructions or ability to adapt the instructions to their style and context.
	Attitudes	Teachers' attitude towards new material (change), science, critical thinking and independent thinking by pupils (or their role as authorities in the classroom).
	Beliefs	Teachers' beliefs about the teaching methods or content (eg, what treatments work or the concepts).
	Emotions	Teachers' emotions, such as stress or anxiety.
	Motivation	Teachers' motivation to teach the material.
	Positive learning environment	Teachers' ability to create a positive learning environment; for example, encourage discussion, respond positively to questions, engage pupils.
Learners	Literacy	Learners' ablity to understand the material.
	Attendance	Learners' attendance or reasons for poor attendance (eg, long distance to school or inability to pay school fees).
	Motivation to learn	Learners' motivation to learn the new material.
	Attitudes	Learners' attitudes towards learning, towards authorities, towards science, towards critical thinking.
	Beliefs	Learners' beliefs about the content (eg, what treatments work or the concepts).
	Home environment	The extent to which the learners's home environment encourages or discourages learning from the lessons.
	Differentiated instruction	The extent to which learners different learning needs are met.
	Peer influence	Positive or negative attitudes of other learners towards the material.
Teaching materials	Value of the material	The extent to which the materials are valued by the teachers and learners.

Supplement to: Ssenyonga R, Lewin S, Nakyejwe E, et al. Process evaluation of teaching critical thinking about health using the Informed Health Choices Intervention in Uganda: a mixed methods study. *Glob Health Sci Pract*. 2024;12(6):e2300484. https://doi.org/10.9745/GHSP-D-23-00484

**Domain**	**Factors and sources**	**Explanation**
	Compatibility with the curriculum	The extent to which the material fits with the rest of the curriculum and how it is taught.
	Appropriateness of the material	The extent to which the materials are relevant, challenging and engaging.
	Credibility of the material	The extent to which the teachers and learners perceive the material as credible.
	Introduction to the lessons	The extent to which the materials were introduced to the schools and learners.
	ICT competencies	The extent to which the teachers easily accessed and used the digital materials to prepare and teach.
School system and environment	Time constraints	The extent to which there is sufficient time to accommodate introducing the new material.
	Competing priorities	The extent to which other priorities for the school, teachers or pupils limit introducing the material (eg, preparing for exams).
	School organisation and management	The extent to which the school provides an environment that supports adoption of new subjects, material and teaching methods.
	School resources, particularly human resources	The extent to which the school has adequate resources to introduce the new materials (eg, human resources, student/teacher ratio, teacher workload, classroom space and classroom resources, such as blackboards and acoustics).
	Attitudes and beliefs of head teacher and other teachers	Attitudes or beliefs of colleagues that influence the teacher's interest in and ability to teach the material.
	Parent and community involvement	Parents' attitudes towards the new material or how things are done at the school.
	Regulation	Regulations (eg, Ministry of Education policies and regulations) that affect introducing the new material.
	Political environment	Elements of the political environment that affect introducing the new material; for example, authoritarianism or teacher strikes.
	Bureaucracy	Bureaucratic arrangements that delay or limit introduction of the new materials, or facilitate introducing them.
	Incentives and disincentives	Incentives or disincentives to introduce the new materials for teachers or head teachers.

Supplement to: Ssenyonga R, Lewin S, Nakyejwe E, et al. Process evaluation of teaching critical thinking about health using the Informed Health Choices Intervention in Uganda: a mixed methods study. *Glob Health Sci Pract*. 2024;12(6):e2300484. https://doi.org/10.9745/GHSP-D-23-00484

Framework for potential adverse (reported in another article) and beneficial effects

**Potential adverse effects**	**Corresponding beneficial effects**
Conflict between children and teachers due to children challenging their teachers	More open and engaging discussion of the basis of diverse claims or beliefs.
Conflict between children and parents due to children challenging their parents	Better understanding between children and parents due to children conversing with their parents about what they are learning and parents feeling more engaged with what their children are learning and engagement of parents in discussions of health issues.
Distrust of health professionals or conflict between children and health professionals	Appropriate questioning of health professionals, better understanding and better healthcare.
Conflict due to undermining of religious beliefs	Engagement of children and others in discussion about religious beliefs and science.
Shortened enjoyment of the innocence of childhood	Increased enjoyment of school and childhood.
Nihilism or cynicism	Healthy scepticism and appreciation science.
Other potential beneficial effects	
Impacts on teachers	The learning resources might improve the teachers' understanding and ability to apply the concepts being taught to the children.
Impacts on parents	The learning resources might indirectly improve parents' understanding and ability to apply the concepts being taught to the children.
Assertiveness	Children asking more questions and not taking things for granted.
Improved decision-making	Children making more thoughtful and informed decisions.
Nonviolent conflict resolution	Claims being presented and addressed in a friendly manner even when there is a disagreement about the claim, as illustrated in the resources.
Friendship formation	Friendly interactions between adults and children and among children, as illustrated in the resources.
Collaboration for problem solving	Collaboration for problem solving among the children, as illustrated in the resources.
Creativity	Thinking outside the box.

Supplement to: Ssenyonga R, Lewin S, Nakyejwe E, et al. Process evaluation of teaching critical thinking about health using the Informed Health Choices Intervention in Uganda: a mixed methods study. *Glob Health Sci Pract*. 2024;12(6):e2300484. https://doi.org/10.9745/GHSP-D-23-00484

**Potential adverse effects**	**Corresponding beneficial effects**
Numeracy	Improvements in numeracy, reflecting what is learnt in lessons 6 and 7 (on fair comparisons and the play of chance).

SUPPLEMENT 4. Team-reflexive methods and statement

Background

Reflexivity involves researchers reflecting on and communicating their *a priori* values, views, experiences and beliefs about the topic of interest, as well as their context, and how they might influence the research (1, 2). Researchers often apply reflexivity individually, but there are benefits to also considering how the dynamics, structure and expectations of the research team may influence the research (3). In a team-reflexive process, members of the research team can discuss how their individual and collective perspectives, beliefs, and experiences might influence or have influenced the design and or conduct of the research, or their interpretation of the findings. This discussion can inspire reflections and facilitate constructive questioning of each other's assumptions. Our objective in undertaking a group reflexivity exercise was to identify important issues to consider and address in the analysis and reporting of the study findings. These are reported in a reflexivity statement herein.

Methods

Written reflections from the team

MO, SL, and AO developed and circulated four open-ended, reflexive questions to members of the research team (FC, RS, SL, AN, DS, SR, JM, AO, AF, NS, MM, MO, MK, AF) and asked them to write their responses. MO, SL, and AO also submitted responses. The questions were:

- What findings do I expect to come out of the process evaluations?
- How do I anticipate that the findings will contribute to the CHOICE project overall?
- How might I shape the process evaluations or my views of them, based on my beliefs (e.g., about the impacts of the intervention); background and previous research experiences (e.g., my disciplinary training); or hopes or concerns related to the CHOICE project?
- What are my concerns related to the CHOICE project, if any?

Research assistants were not included in this stage in order to keep the amount of feedback manageable. AN coded and analysed the written responses using thematic analysis methods (4).

Team discussions

All team members were invited to a structured team-reflexive discussion (FC, RS, SL, AN, DS, SR, JM, AO, AF, NS, MM, MO, MK, AF) in January 2023 during a period where researchers were engaged in data analysis for the process evaluations. The discussion lasted two hours and was facilitated by HMK. Findings from the analysis of the written reflections informed a discussion guide focused on the following themes:

- What findings do I expect to come out of the process evaluations?
- How might I shape the process evaluations or my views of them, based on my beliefs (e.g., about the impacts of the intervention); background and previous research

experiences (e.g., my disciplinary training); or hopes or concerns related to the CHOICE project?

- How do I anticipate that the findings will contribute to the CHOICE project overall?

The research team members were invited to a second structured team-reflexive discussion in April 2023 (FC, RS, SL, AN, DS, SR, JM, AO, AF, NS, MM, MO, MK, AF). HMK and MO prepared the discussion guide based on on the topics covered in the first team discussion. HMK facilitated the discussion around the following themes:

- What are other concerns aside from implementation and sustainability (covered during first discussion)?
- Where do these concerns come from?
- What are the relationships between the project team members and how does that impact the research?

Parts of the first team discussion were recorded. The second team discussion was recorded, but the recording was destroyed before transcription. Two people observed and took notes for each discussion.

HMK drafted a team-reflexive statement with key issues that we identified and discussed. All authors agreed on the final statement.

HMK had responsibility for planning and leading the discussions, and drafting the statement, because she did not contribute to the development of the intervention, nor to planning or conducting the evaluation.

Analysing our reflections

HMK used framework analysis to identify themes from the team discussions (5). HMK combined the analyses from the written reflections and group discussions using thematic analysis (4). The following is a reflexivity statement based on the results of the written responses and two structured team-reflexive discussions.

Reflexivity Statement

Background of researchers

The research team consisted of 16 researchers who represent a wide array of methodological experience, involvement in the Informed Health Choices project, and geographic and cultural backgrounds. Most of the researchers (SL, AN, DS, SR, JM, AO, AF, NS, MM, MO, MK, AF) were involved in the development of Informed Health Choices primary school intervention, as well as the secondary school intervention (6). Four of the researchers are leading various components of the CHOICE project as part of their doctoral work (FC, MM, RS, MO). None of the researchers are teachers, educational specialists, curriculum developers, or otherwise involved with the development or implementation of school curricula.

The team consisted mostly of researchers with health-related backgrounds. It may have been advantageous to have included researchers with more expertise in education and curriculum development when designing the studies and interpreting the findings. However, we did seek feedback from a wide variety of stakeholders—such as education researchers—at all stages of the project.

Research team members who were responsible for engaging with stakeholders, collecting feedback during intervention development, as well as implementing and evaluating the intervention had relevant geographical and cultural backgrounds. This in-depth understanding of the research contexts may have improved the design of the educational resources and the conduct of the studies.

Expectations regarding the process evaluation findings

Most of the research team expected positive findings from the process evaluations. However, all team members also expected important implementation and sustainability challenges to emerge from the process evaluation, and some team members expected findings of potential adverse effects of the intervention to emerge. These expectations may have influenced how the findings were interpreted.

Concerns regarding the intervention and evaluation

Effects, scale-up, and sustainability of the intervention

Within the team there were varying perspectives regarding whether or how the intervention could be implemented beyond the research project. Given that these views varied, it is unlikely that they influenced the analysis of the findings.

Scope of the evaluation

The research team may have viewed the findings only within the scope of the project and not sufficiently explored how this project fits in with, or could be enhanced by, other research in the field. On the other hand, the practice approach used in this project allowed for identifying issues that could be addressed and improved upon in further research.

Researchers' relationship to the project and to the participants

The review team was responsible for both developing and evaluating (collecting data for) the intervention. This may have hindered honest and critical feedback from the research participants (e.g., teachers) in that they did not want to offend or disappoint the researchers. It may also have influenced how the research team has analysed the data: since they were responsible for developing the resources, they may interpret results in a more positive manner.

Dynamics within the research team

Team members' responsibilities (e.g., leading process evaluations or a review of adverse effects) could have influenced their interpretation of the data from the process evaluation. For example, some members could have leaned towards overstating positive findings and overlooking negative findings due to the time, energy, and resources they had invested in developing the intervention.

Supplement to: Ssenyonga R, Lewin S, Nakyejwe E, et al. Process evaluation of teaching critical thinking about health using the Informed Health Choices Intervention in Uganda: a mixed methods study. *Glob Health Sci Pract*. 2024;12(6):e2300484. https://doi.org/10.9745/GHSP-D-23-00484

Given that the study took place largely during the COVID-19 pandemic, there were limited opportunities in the earlier phases of the project for face-to-face meetings or team-building events, within and especially across countries. Some team members noted that this may have impacted on team dynamics. During the last phase of the project (data analysis and planning the 1-year follow up) many of the project team members from different countries met regularly face-to-face, but in Europe (SR, JM, AO, MO), not the East-African contexts in which we conducted the research.

References

1. Downe S, Finlayson KW, Lawrie TA, Lewin SA, Glenton C, Rosenbaum S, et al. Qualitative evidence synthesis (QES) for guidelines: paper 1–Using qualitative evidence synthesis to inform guideline scope and develop qualitative findings statements. Health research policy and systems. 2019;17(1):1-12.
2. Olmos-Vega FM, Stalmeijer RE, Varpio L, Kahlke R. A practical guide to reflexivity in qualitative research: AMEE Guide No. 149. Medical teacher. 2022:1-11.
3. Barry CA, Britten N, Barber N, Bradley C, Stevenson F. Using reflexivity to optimize teamwork in qualitative research. Qualitative health research. 1999;9(1):26-44.
4. Braun V, Clarke V. Thematic analysis: American Psychological Association; 2012.
5. Ritchie J, Spencer L, O'Connor W. Carrying out qualitative analysis. Qualitative research practice: A guide for social science students and researchers. 2003;2003:219-62.
6. Nsangi A, Semakula D, Rosenbaum SE, Oxman AD, Oxman M, Morelli A, et al. Development of the informed health choices resources in four countries to teach primary school children to assess claims about treatment effects: a qualitative study employing a user-centred approach. Pilot and Feasibility Studies. 2020;6(1):1-15.

Supplement to: Ssenyonga R, Lewin S, Nakyejwe E, et al. Process evaluation of teaching critical thinking about health using the Informed Health Choices Intervention in Uganda: a mixed methods study. *Glob Health Sci Pract*. 2024;12(6):e2300484. https://doi.org/10.9745/GHSP-D-23-00484

SUPPLEMENT 5. GRADE-CERQual qualitative evidence profile

In the table below describe what concerns, if any, do you have about each component. Indicate your level of concern as: **no or very minor, minor**, **moderate** or **serious.**

Below is each component assessed to make an overall GRADE-CERQual assessment of confidence in the finding scored as: **high**, **moderate**, **low** or **very low**), and explanation for the assessment.

	Finding	Sources	Methodological limitations	Coherence	Adequacy	Relevance	Confidence	Explanation
1	All the students, teachers, head teachers and policy makers interviewed valued the lessons and recognised their importance.	FDGs, key informants (teachers, head teachers, policy makers)	No concerns	No concerns. There was coherence of the finding across the two sources of data.	Rich data from all participants	No concerns.	High	No concerns regarding methodological limitations, relevance, coherence, or adequacy
2	Nearly all planned IHC lessons were taught. Recommended teaching strategies and anticipated time for preparation (30 minutes) were used. However, few lessons were delivered as scheduled (once a week, during normal class time and within 40 minutes). Most lessons were taught in students' private reading time, and all took longer than 40 minutes to deliver.	FGDs, lesson evaluation forms, observation forms	No concerns	No concerns. There was coherence of the finding across the four sources of data.	Sufficiently rich data from many participants	No concerns.	High	No concerns regarding methodological limitations, relevance, coherence, or adequacy
3	Finding time to teach the IHC lessons impeded delivery as intended and may also affect scale up of the intervention. Factors such as a busy school term that included sports, drama, and patriotic clubs meant moving lessons around, often teaching two IHC lessons in one week and not once a week as planned. There also was a teachers' school strike the first three weeks of the term.	FGDs key informants (Teachers), lesson evaluation forms, observation forms	No concerns	No concerns. There was coherence of the finding across the four sources of data.	Sufficiently rich data from many participants	No concerns	High	No concerns regarding methodological limitations, relevance, coherence, or adequacy

Supplement to: Ssenyonga R, Lewin S, Nakyejwe E, et al. Process evaluation of teaching critical thinking about health using the Informed Health Choices Intervention in Uganda: a mixed methods study. *Glob Health Sci Pract*. 2024;12(6):e2300484. https://doi.org/10.9745/GHSP-D-23-00484

	Finding	Sources	Methodological limitations	Coherence	Adequacy	Relevance	Confidence	Explanation
4	Because the IHC lessons were not in the curriculum and not nationally examined, teachers' preparation for lessons, students' attendance, and head teachers' prioritisation of the lessons were limited. Head teachers must decide what can be taught that contributes to the students' scores. Teachers are often acknowledged based on their students' performance on national examinations. Students take additional learning materials that are not examinable less seriously	FGDs, key informants (students, teachers, head teachers, policy makers), observation forms	No concerns	No concerns. There was coherence of the finding across the two sources of data.	Sufficiently rich data from many participants	No concerns	High	No concerns regarding methodological limitations, relevance, coherence, or adequacy
5	Students found the lessons enjoyable, understandable (simple English, familiar examples) and related to the health issues that the lessons addressed.	FGDs, key informants (students, teachers), lesson evaluation forms, observation forms	No concerns	No concerns. There was coherence of the finding across the four sources of data.	Sufficiently rich data from many participants	No concerns	High	No concerns regarding methodological limitations, relevance, coherence, or adequacy
6	Teachers found the IHC resources easy to access and adaptable (similar teaching strategies to those in the new curriculum, lesson structure easy to follow).	Workshop evaluation forms, key informants (teachers, curriculum developers), lesson evaluation forms	No concerns	No concerns. There was coherence of the finding across the three sources of data.	Sufficiently rich data from many participants	No concerns	High	No concerns regarding methodological limitations, relevance, coherence and adequacy.
7	Students were able to understand and apply the key concepts. Several students gave illustrations of the concepts and how they applied them to think critically about health. This was mainly for concepts about claims and less for concepts about research.	FDGs, key informants (students, teachers, head teachers, parents)	No concerns	No concerns. There was coherence of the finding across the two sources of data.	Sufficiently rich data from many participants	No concerns	High	No concerns regarding methodological limitations, relevance, coherence, or adequacy
8	Students expressed increased interest in STEM subjects (biology, physics, chemistry, ICT and math) and the health profession. Teachers also noticed this interest among some of their students.	FDGs, key informants (students, teachers)	No concerns	No concerns. There was coherence of the finding across the two sources of data.	Minor concerns. Moderately rich data from many participants.	No concerns	Moderate	Minor concerns regarding adequacy

Supplement to: Ssenyonga R, Lewin S, Nakyejwe E, et al. Process evaluation of teaching critical thinking about health using the Informed Health Choices Intervention in Uganda: a mixed methods study. *Glob Health Sci Pract*. 2024;12(6):e2300484. https://doi.org/10.9745/GHSP-D-23-00484

	Finding	Sources	Methodological limitations	Coherence	Adequacy	Relevance	Confidence	Explanation
9	Teacher training improved understanding, motivation, and confidence among teachers to deliver the IHC lessons.	Workshop evaluation forms, key informants (teachers)	No concerns	No concerns. There was coherence of the finding across the two sources of data.	Sufficiently rich data from many participants	No concerns	High	No concerns regarding methodological limitations, relevance, coherence, or adequacy
10	Credibility of the institution that developed the resources may have impacted effective delivery. Some student and teachers mentioned that they viewed material from Makerere University as important to learn. Other teachers mentioned the presence of curriculum developers at the teacher training workshop improved their trust of the resources.	FDGs, key informants (students, teachers), workshop evaluation forms	No concerns	No concerns. There was coherence of the finding across the three sources of data.	Sufficiently rich data from many participants	No concerns	High	No concerns regarding methodological limitations, relevance, coherence, or adequacy
11	Some policymakers and teachers expressed the desire to have printed materials alongside the digital materials, particularly if the intervention was to be scaled up. For some, this was due to ICT challenges and already existing provisions of printed materials in the new curriculum. For others, this was to give students easy access to the material.	FDGs, key informants (teachers, policymakers, and a few students)	No concerns	No concerns	Data from four participants. Some explanation provided for their views. Limited data from students.	No concerns	Moderate	Minor concerns about adequacy
